# Prediction model of acute kidney injury after different types of acute aortic dissection based on machine learning

**DOI:** 10.3389/fcvm.2022.984772

**Published:** 2022-09-21

**Authors:** Li Xinsai, Wang Zhengye, Huang Xuan, Chu Xueqian, Peng Kai, Chen Sisi, Jiang Xuyan, Li Suhua

**Affiliations:** ^1^Kidney Disease Center of the First Affiliated Hospital of Xinjiang Medical University, Urumqi, China; ^2^Xinjiang Branch of National Clinical Research Center for Kidney Disease, Institute of Nephrology of Xinjiang, Urumqi, China; ^3^Xinjiang Blood Purification Medical Quality Control Center, Urumqi, China; ^4^School of Public Health, Xinjiang Medical University, Urumqi, China

**Keywords:** acute renal injury, machine learning, prediction model, acute aortic dissection, Type A acute aortic dissection, Type B acute aortic dissection

## Abstract

**Objective:**

A clinical prediction model for postoperative combined Acute kidney injury (AKI) in patients with Type A acute aortic dissection (TAAAD) and Type B acute aortic dissection (TBAAD) was constructed by using Machine Learning (ML).

**Methods:**

Baseline data was collected from Acute aortic division (AAD) patients admitted to First Affiliated Hospital of Xinjiang Medical University between January 1, 2019 and December 31, 2021. (1) We identified baseline Serum creatinine (SCR) estimation methods and used them as a basis for diagnosis of AKI. (2) Divide their total datasets randomly into Training set (70%) and Test set (30%), Bootstrap modeling and validation of features using multiple ML methods in the training set, and select models corresponding to the largest Area Under Curve (AUC) for follow-up studies. (3) Screening of the best ML model variables through the model visualization tools Shapley Addictive Explanations (SHAP) and Recursive feature reduction (REF). (4) Finally, the pre-screened prediction models were evaluated using test set data from three aspects: discrimination, Calibration, and clinical benefit.

**Results:**

The final incidence of AKI was 69.4% (120/173) in 173 patients with TAAAD and 28.6% (81/283) in 283 patients with TBAAD. For TAAAD-AKI, the Random Forest (RF) model showed the best prediction performance in the training set (AUC = 0.760, 95% CI:0.630–0.881); while for TBAAD-AKI, the Light Gradient Boosting Machine (LightGBM) model worked best (AUC = 0.734, 95% CI:0.623–0.847). Screening of the characteristic variables revealed that the common predictors among the two final prediction models for postoperative AKI due to AAD were baseline SCR, Blood urea nitrogen (BUN) and Uric acid (UA) at admission, Mechanical ventilation time (MVT). The specific predictors in the TAAAD-AKI model are: White blood cell (WBC), Platelet (PLT) and D dimer at admission, Plasma The specific predictors in the TBAAD-AKI model were N-terminal pro B-type natriuretic peptide (BNP), Serum kalium, Activated partial thromboplastin time (APTT) and Systolic blood pressure (SBP) at admission, Combined renal arteriography in surgery. Finally, we used in terms of Discrimination, the ROC value of the RF model for TAAAD was 0.81 and the ROC value of the LightGBM model for TBAAD was 0.74, both with good accuracy. In terms of calibration, the calibration curve of TAAAD-AKI's RF fits the ideal curve the best and has the lowest and smallest Brier score (0.16). Similarly, the calibration curve of TBAAD-AKI's LightGBM model fits the ideal curve the best and has the smallest Brier score (0.15). In terms of Clinical benefit, the best ML models for both types of AAD have good Net benefit as shown by Decision Curve Analysis (DCA).

**Conclusion:**

We successfully constructed and validated clinical prediction models for the occurrence of AKI after surgery in TAAAD and TBAAD patients using different ML algorithms. The main predictors of the two types of AAD-AKI are somewhat different, and the strategies for early prevention and control of AKI are also different and need more external data for validation.

## Introduction

Acute kidney injury is one of the major complications of Cardiac and vascular surgery (CVS), which has been named CVS-AKI by scholars (1). AAD is a typical representative of acute and critical cardiovascular disease. In recent years, the detection rate of aortic coarctation has been increasing year by year with the rise of public health awareness and the improvement of medical treatment. Postoperative AKI leads to increased length of stay and costs for patients and is considered an important factor in poor outcomes, including death, and is a focal point that needs to be addressed urgently (2–7). The AAD is currently divided into two types, A and B, according to the location of the rupture, using the Stanford typing. TAAAD opens in the ascending aorta and often tears proximally or distally, making the attack extremely dangerous and often requiring urgent surgical repair. The incidence of TAAAD- AKI reports ranged widely from 26 to 72% (3, 6, 8–15). Patients with TBAAD involving only the descending aorta and poorly treated with conservative medications often require angiographic guidance for EVAR (endovascular aortic repair). The incidence of AKI after TBAAD decreased compared to TAAAD, but still ranged from 17.9 to 52.7% (2, 5, 7, 16). The treatment of AKI currently lacks specific drugs, so early identification and intervention remains a current hot topic in AKI research.

In recent years, many predictive models for AKI after cardiac surgery have been investigated and developed in China and abroad. Includes Cleveland prediction model requiring RRT (17), Mehta prediction model (18), Pannu predictive model (19) and MCSPI prediction models without RRT (20), Chuang WN prediction model (21) etc. However, most of the above models are based on heart valve or coronary surgery, lack prediction models for AAD-AKI, and mostly use Logistic Regression (LR) methods that do not solve the nonlinearity problem well to construct models. ML methods developed in recent years have been used to improve the performance of clinical prediction (22). Since the risk level of TAAAD and TBAAD and the surgical approach are completely different, the mechanism and degree of AKI occurring after surgery and its prognosis are also very different. Therefore, this study constructs clinical prediction models for each of the two types of AAD-AKI based on machine learning methods to screen the characteristic variables and provide a basis for early prevention and intervention of AKI in AAD patients.

## Methods

### Study population

We retrospectively investigated 1,543 patients with AAD (of which all TAAAD were from our thoracic surgery department for open vascular replacement surgery; TBAAD were from vascular surgery or cardiology department for EVAR) who were hospitalized and treated with surgery from January 1, 2019 to December 30, 2021 at the First Affiliated Hospital of Xinjiang Medical University. Exclusion criteria: (1) patients who had already started Renal Replacement Therapy (RRT) or died before surgery; (2) patients with intermural hematoma and simple aortic aneurysm; (3) patients with missing SCR and incomplete relevant clinical data. The data of 456 patients were finally included by excluding 1,087 cases who were not eligible. Among them, 173 (38%) were TAAAD patients and 283 (62%) were TBAAD patients.

### Data collection

**Figure 1:**
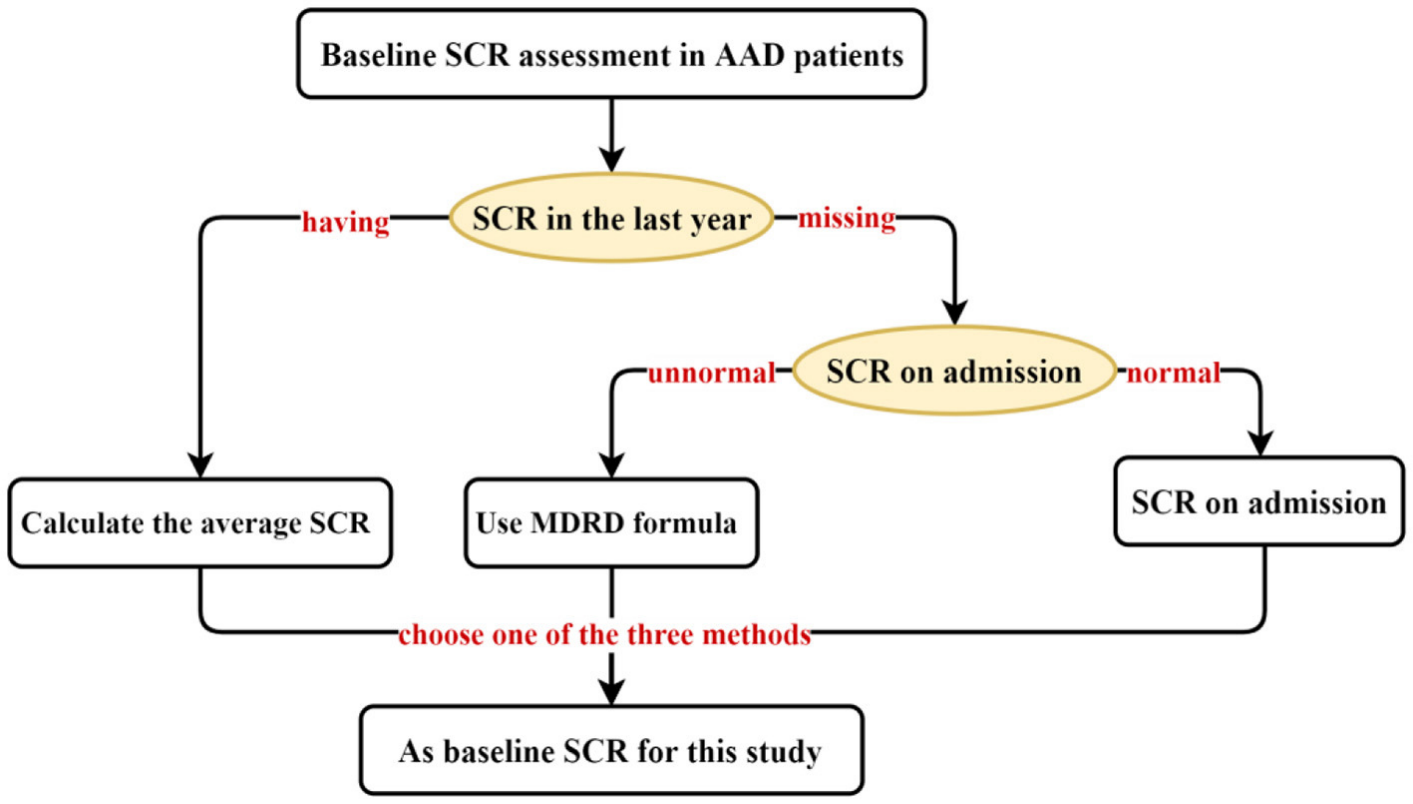
Baseline creatinine assessment method for patients with AAD.

We collected data on demographic characteristics, vital signs, common comorbidities, preoperative, intraoperative, and postoperative indicators, and perioperative medication regimens of hospitalized AAD patients through Electronic Medical Record (EMR). Ultimately, 46 characteristics were included for TAAAD patients and 42 characteristics were used by TBAAD to construct the initial model for, respectively. AKI is diagnosed using the 2012 Kidney Disease: Improving Global Outcomes (KDIGO) Clinical Practice Guidelines for Acute Kidney Injury (23), That is, SCR increased by 26.5 μmol/L (0.3 mg/dL) within 48 h, or increased by 50% from baseline SCR within 7 d, or persisted for more than 6 h with urine output <0.5 mL/(kg·h). In fact, there is no definitive standard for baseline SCR. For patients lacking a stable and reliable baseline SCR record, the Risk, Injury, Failure, Loss of Kidney Function, and End-stage Kidney Disease (RIFLE) (24) and The KDIGO guidelines recommend using the Modification of Diet in Renal Disease (MDRD) formula to retrograde the baseline SCR. The MDRD formula uses variables such as SCR, age, sex, and race to estimate the glomerular filtration rate (GFR) of the kidney (25). While the European Renal Best Practice (ERBP) recommends using the admission SCR (26), most investigators in clinical practice mostly follow the ERBP guidelines. However, Siew et al. (27) found that this approach resulted in an underestimation of AKI incidence by 46%. In addition, one study (28) found that in patients without predominantly nephropathy, the mean serum SCR at one-year outpatient follow-up was closest to their true GFR. We combined the guideline recommendations and the characteristics of AAD onset and selected the optimal assessment method from three perspectives as the baseline SCR for patients with AAD (Figure 1).

### Machine learning

We tried the following most popular supervised machine learning methods to develop prediction models for classification outcomes: DT (Decision Tree), RF, XGboost (eXtreme Gradient Boosting), and LightGBM, and compared them with traditional LR methods. These ML models are closer to the human mind and have high interpretability as well as high accuracy. The DT method is the simplest tree model (29). The elements that make it up are nodes and edges, where the nodes are judged based on the various characteristics of the samples and the edges refer to the next classification direction DT uses the idea of top-down recursion to construct information entropy falling fastest tree, and uses Gini impurity as an indicator for classification result judgment. The Gini index is the probability of random classification error of samples in the data set. However, simple DT has the disadvantages of overfitting and weak generalization ability, so RF is derived on this basis. The basic idea of RF is to construct the final prediction model by constructing decision trees of random samples several times and using bagging to output the results in a voting way (30). XGboost and LightGBM are optimizations of Gradient Boosting Decision Tree (GBDT), an iterative decision trees algorithm that uses weak classifiers to make decision trees accumulate predictions that are closer to the true value by continuously reducing the residuals of the model. The model is used to reduce the residuals so that the predictions accumulated by the decision trees are closer to the true values. While XGboost (31) mainly adds regularization to GBDT to build penalty function to reduce overfitting. lightGBM uses a Histogram (histogram) based decision trees algorithm and uses a Leaf-wise (leaf growth) strategy with maximum splitting gain, which is more efficient in terms of training speed. It has a significant advantage in handling large data in particular (32). Each of these methods has its own advantages and disadvantages in different dataset environments, and there is no absolute advantage or disadvantage.

### Statistical analysis

The Python software version 3.7.13 (https://www.python.org) was used for statistical processing and analysis, mainly including the drawing toolkit Matplotlib (version 3.3.4), and the machine learning framework scikit-learn (versions 0.24.2 and 1.0. 2). In the present study, for continuous variables, the Kolmogorov-Smirnov test was used to assess the normal distribution of the data when performing the characterization, and $\bar{x}$ ± s was used for measures that obeyed normality, and *t*-tests were used to compare between groups in the training and test sets. Variables that did not follow a normal distribution were described using M (P25,P75), and group comparisons were made using the Wilcoxon rank sum test for two independent samples. For categorical variables, n (%) was used for description and group comparisons were made using the χ^2^ test. A two-tailed test was set and *p* < 0.05 was statistically different.

For both TAAAD and TBAAD datasets, we randomly divided them into a training set (70%) and a test set (30%). Using Logistic Regression and various ML methods in the training set, we use Bootstrap resampling technique (1,000 times) to randomly select a random number of samples in the training set each time for initial modeling, and the unsampled part for internal validation, and evaluate the prediction accuracy by plotting the receiver operating characteristic (ROC) curve of each model in the training set and calculating the AUC size to evaluate the prediction accuracy, and the best performing model is used for subsequent studies.

To analyze the specific contributions made by the features included in this study to the model, we used the feature visualization toolkit Shap (version 0.40.0) for observing the specific performance of features in the model, which is based on the idea of game theory and belongs to a *post hoc* explanatory framework that can provide specific Shapley values to assess the importance of each feature in each sample relative to the target variable (33). In addition, we filtered the variables through a method based on RFE from the Sklearn library, a greedy algorithm for finding the optimal subset of features, which is more stable in dealing with multicollinearity problems than the previously used Lasso regression. The variables are filtered using a five-fold cross-validation to output stable and reliable variables, and the final variables for inclusion in the compact prediction model are determined by the results of SHAP and RFE. In addition, hyperparametric search and optimization of the screened prediction models are performed using the GridSearch method in the Sklearn library.

**Figure 2:**
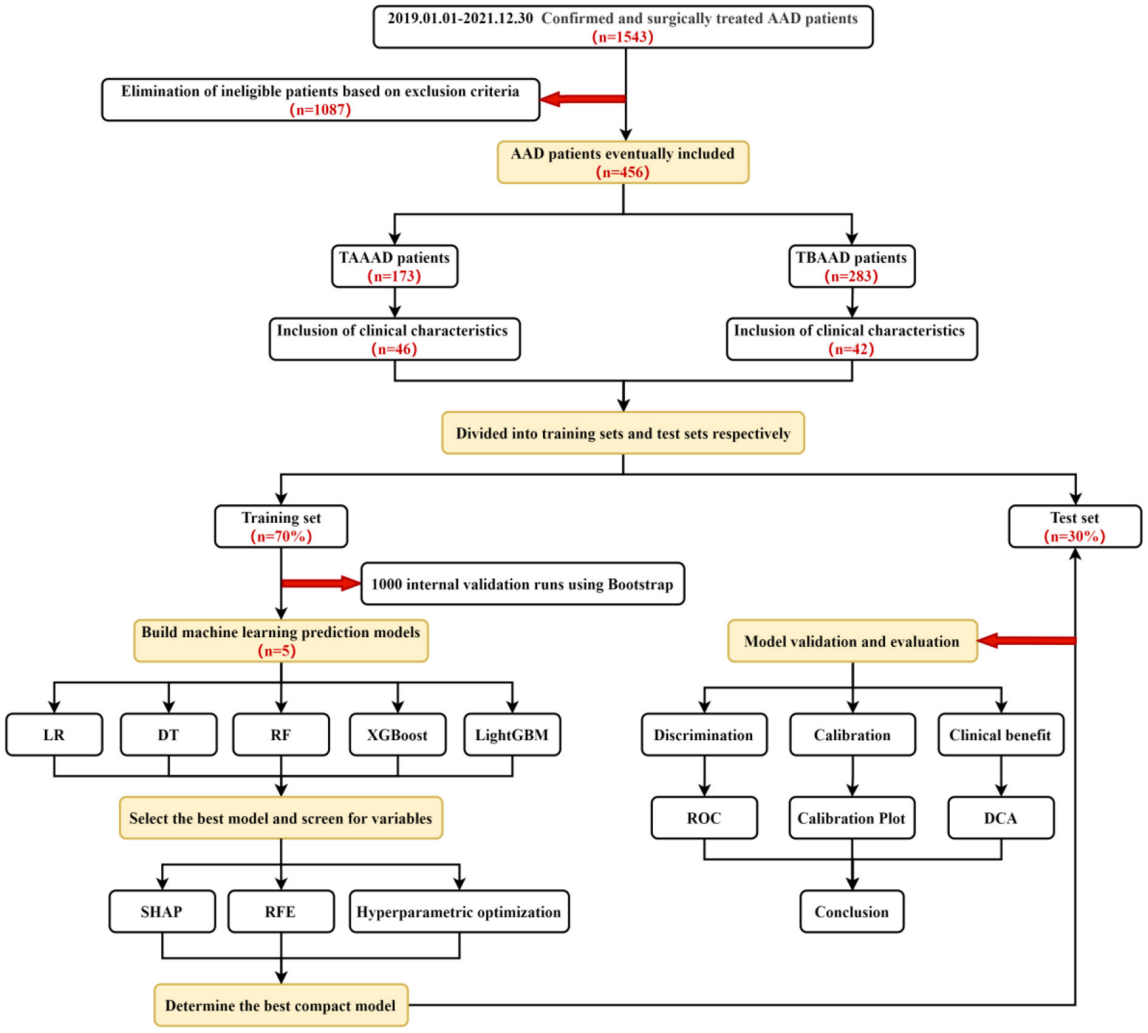
Technology roadmap.

Finally, we evaluated the final incorporated compact machine models in terms of three dimensions: Discrimination, Calibration and Clinical benefit using the test set data. In terms of discrimination, we compared the performance of the compact models with the widely used clinical metrics SCR, BUN and UA in diagnosing AKI by plotting the ROC curves in the test set. In addition, to evaluate the difference between the predicted and true results of each machine learning model for AKI occurrence, we plotted a calibration plot in the test set, where the *x-*axis of the curve represents the predicted probability of AKI and the *y-*axis represents the actual probability of AKI occurrence. The dashed line represents the ideal curve, and the closer to the dashed line, the higher the agreement. In addition, the Brier score is used to quantitatively assess the agreement, and the lower the Brier score for a set of predicted values, the better the prediction calibration. To show the practical utility of the model in the clinic, we also plotted the DCA curve to meet the practical needs of clinical decision makers. The *X-*axis of this curve is the threshold probability and the *Y-*axis is the net benefit corresponding to each threshold probability (Figure 2).

## Results

### Baseline characteristics

**Figure 3:**
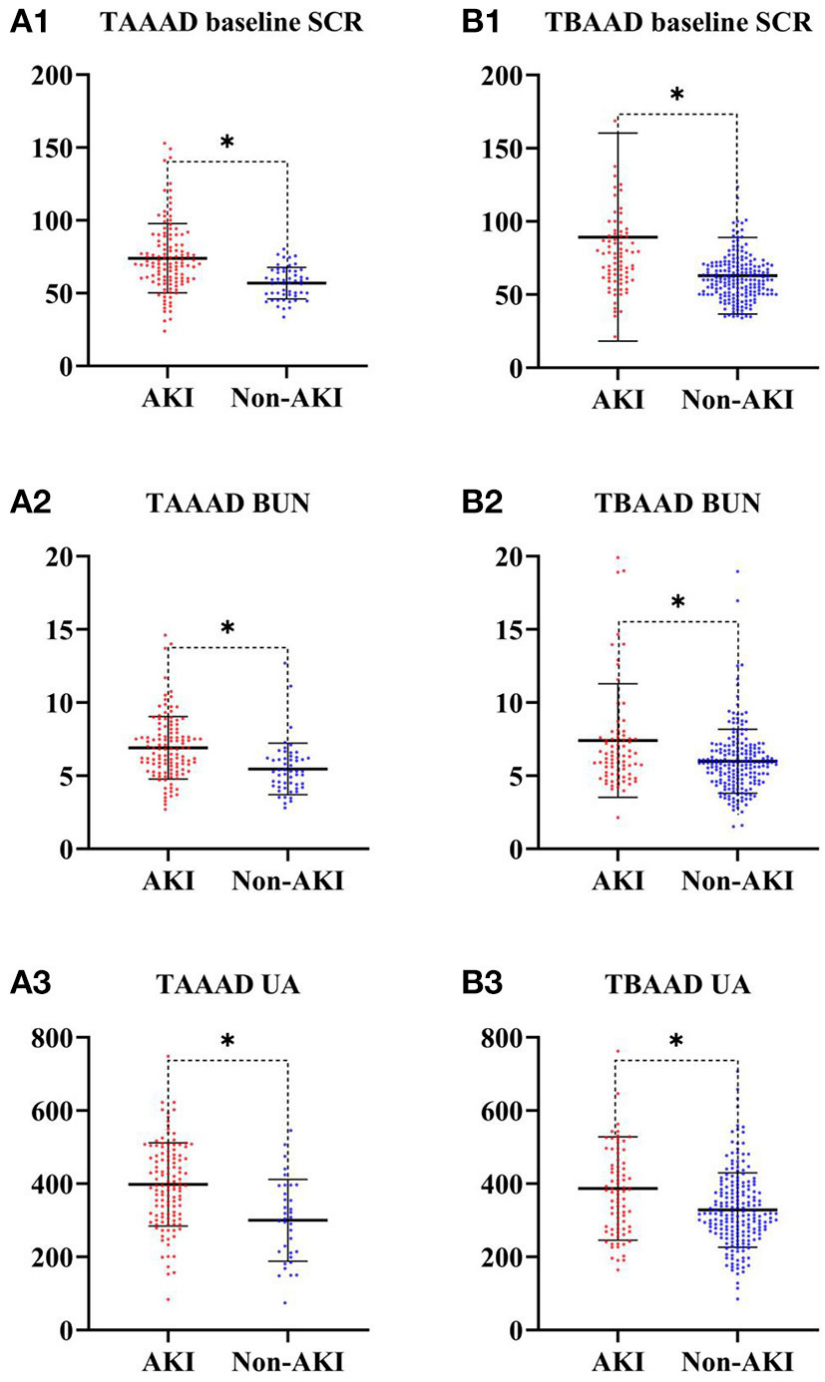
SCR, BUN and UA differences between AKI and Non-AKI groups. ^*^ represents statistical differences. **(A)** Is from the TAAAD data; **(B)** is from the TBAAAD data.

In our research, the incidence of postoperative AKI was 69.4% (120/173) in TAAAD patients and 28.6% (81/283) in TBAAD patients, both slightly higher than in other studies (8, 16, 34). SCR, BUN and UA are currently the most commonly used clinical indicators to respond to kidney function (35), but these metabolites are not markers of kidney injury and may be interfered with by other factors such as feeding and metabolism. We performed a univariate analysis of these two most sensitive indicators for the diagnosis of AKI, and Figure 3 demonstrates that the differences in baseline SCR, admission BUN and UA in the total data set for both types of AAD were statistically significant (*p* < 0.05) in both the AKI and non-AKI groups. This study depicts the concentration and dispersion of each variable in the total data set for patients with both types of AAD, and furthermore, the total data set was randomly split into a training set and a test set by 7:3, and all variables did not differ significantly in both split data sets. This indicates that the training and test set data are homogeneous and comparable (see Tables 1, 2).

**Table 1:** TAAAD patient characteristics and perioperative variables.

**TAAAD variables**	**All** **(*N =* 173)**	**Training set** **(*N =* 121)**	**Test set** **(*N =* 52)**	***P***
Age (years)	48.53 ± 8.47	48.54 ± 8.69	48.52 ± 8.03	0.99
Male, *n* (%)	150(86.70)	105(86.78)	45(86.53)	0.84
**Preoperative factors**				
Hypertension, *n* (%)	121(69.94)	83(68.59)	38(73.08)	0.68
Diabetes, *n* (%)	22(12.72)	16(13.22)	6(11.54)	0.95
CCD, *n* (%)	15(8.67)	12(9.92)	3(5.77)	0.55
CKD, *n* (%)	10(5.78)	7(5.78)	3(5.77)	0.720
History of smoking, *n* (%)	71(41.04)	45(37.19)	26(50.00)	0.16
**Renal artery involvement in CTA**				0.34
No, *n* (%)	105(60.69)	77(63.64)	28(53.85)	
Unilateral, *n* (%)	52(30.06)	35(28.92)	17(32.69)	
Bilateral, *n* (%)	16(9.25)	9(7.44)	7(13.46)	
SBP at admission (mmHg)	136.53 ± 24.30	136.64 ± 25.56	136.27 ± 21.33	0.93
DBP at admission (mmHg)	75.85 ± 13.78	76.04 ± 13.97	75.42 ± 13.47	0.79
EF (%)	60.74 ± 4.81	60.69 ± 4.91	60.86 ± 4.61	0.83
WBC (10^9^/L)	13.09 ± 4.36	13.18 ± 4.61	12.88 ± 3.72	0.68
HGB (g/L)	139.81 ± 16.86	139.49 ± 16.78	140.56 ± 17.18	0.70
PLT (10^9^/L)	184.31 ± 71.12	189.26 ± 75.30	172.79 ± 59.33	0.16
APTT (s)	30.9(29.0, 33.7)	31(29.3, 33.8)	30.35(28.7, 33.2)	0.22
D dimer (ng/mL)	2,179(799.0, 3,885.0)	2,051(799.0, 3,605.0)	2,732(811.5, 3,997.5)	0.40
Serum kalium (mmol/L)	3.72 ± 0.51	3.73 ± 0.53	3.71 ± 0.47	0.86
Blood calcium (mmol/L)	2.20 ± 0.12	2.21 ± 0.12	2.20 ± 0.13	0.62
ALT (u/L)	29.55(22.4, 46.0)	29.85(23.0, 49.04)	29.05(20.7, 38.07)	0.44
BUN (mmol/L)	6.47 ± 2.13	6.45 ± 2.19	6.52 ± 2.01	0.83
UA (mmol/L)	373.77 ± 116.96	363.77 ± 125.10	397.02 ± 92.30	0.09
Baseline SCR (umol/L)	75.0(62.55,94.56)	73.24(62.55, 93.8)	77.85(62.72, 97.79)	0.28
Proealcitonin (ng/mL)	0.07(0.04, 0.17)	0.07(0.04,0.17)	0.06(0.05, 0.145)	0.71
IL-6 (pg/mL)	64.7(29.4, 104.4)	64.7(27.31, 115.0)	64.2(32.95, 98.16)	0.97
CTn I (ug/L)	0.024(0.01, 0.16)	0.018(0.01, 0.158)	0.031(0.013, 0.183)	0.35
Pericardial effusion, *n* (%)	45(26.01)	33(27.27)	12(23.08)	0.70
Pleural effusion, *n* (%)	47(27.17)	34(28.10)	7(13.46)	0.81
N-terminal pro BNP (ng/L)	211.0(84.81, 678.0)	241.0(84.81, 691.0)	196.0(89.105, 630.5)	0.60
LAC (mmol/L)	1.7(1.2, 2.7)	1.7(1.3, 2.9)	1.6(1.15, 2.2)	0.06
PaO_2_/FiO_2_ (mmHg)	331.82 ± 128.40	333.61 ± 129.66	327.63 ± 126.57	0.78
**Intraoperative factors**				
Emergency operation, *n* (%)	93(53.76)	64(52.89)	29(55.77)	0.85
CPB duration (mins)	150.0(120.0, 174.0)	150.0(120.0, 176.0)	144.0(112.0, 169.5)	0.41
DHCA (mins)	79.0(68.0, 97.0)	79.0(68.0, 97.0)	77.5(67.5, 95.0)	0.75
Intraoperative bleeding volume (mL)	1,000(1,000.0,1,800.0)	1,000(1,000.0,1,800.0)	1,000(1,000.0,1,600.0)	0.70
Red blood cell transfusion (units)	4.0(3.0, 7.0)	4.0(3.0, 7.0)	4.0(3.0, 6.0)	0.34
Plasma transfusion (mL)	1,760(1,510, 2,180)	1,760(1,510, 2,090)	1,755(1,515, 2,250)	0.99
**Type of operation**				
Ascending aortic or hemiarch replacement, *n* (%)	157(90.75)	109(90.08)	48(92.31)	0.86
Total arch replacement, *n* (%)	125(71.43)	88(72.73)	37(71.15)	0.98
Aortic root replacement, *n* (%)	105(60.69)	70(57.85)	35(67.31)	0.32
Simultaneous coronary artery bypass grafting, *n* (%)	21(12.14)	17(14.05)	4(7.69)	0.88
**Postoperative factors**				
MVT (hours)	199.49 ± 166.81	192.46 ± 163.94	215.85 ± 173.81	0.40
LOS in ICU (days)	12.94 ± 10.37	12.06 ± 9.58	15.00 ± 11.85	0.09
**Perioperative drugs**				
RASI, *n* (%)	67(38.73)	50(41.32)	17(32.69)	0.37
Loop diuretics, *n* (%)	168(97.11)	118(97.52)	50(96.15)	0.99
Vasopressors, *n* (%)	155(89.59)	106(87.60)	49(94.32)	0.30
Statins, *n* (%)	16(9.25)	12(9.92)	4(7.69)	0.86
Antibiotics, *n* (%)	172(99.42)	120(99.17)	52(100)	0.66

CCD, cardia-cerebrovascular disease; CKD, chronic kidney diseases; CTA, computed tomography angiography; SBP, systolic blood pressure; DBP, diastolic blood pressure; EF, ejection fraction; WBC, white blood cell; HGB, hemoglobin; PLT, platelet; APTT, activated partial thromboplastin time; ALT, alanine aminotransferase; AST, aspartate aminotransferase; BUN, blood urea nitrogen; UA, uric acid; SCR, serum creatinine; IL, interleukin; CTn, cardic troponin; BNP, B-type natriuretic peptide; LAC, lactic acid concentration; PaO2, arterial oxygen tension; FiO2, inspired oxygen fraction; CPB, cardiopulmonary bypass; DHCA, deep hypothermic circulatory arrest; MVT, mechanical ventilation time; LOS, length of stay; ICU, intensive care unit; RASI, renin angiotensin aldosterone system inhibitor. Emergency surgery is defined as surgical treatment within 24 hours of admission.

**Table 2:** TBAAD patient characteristics and perioperative variables.

**TBAAD Variables**	**All** **(*N =* 283)**	**Training set** **(*N =* 198)**	**Test set** **(*N =* 85)**	***P***
Age (years)	51.98 ± 11.22	51.40 ± 11.33	53.33 ± 10.90	0.18
Male, *n* (%)	229(80.9)	164(82.8)	20(23.5)	0.28
**Preoperative factors**				
Hypertension, *n* (%)	248(87.6)	175(88.4)	73(85.9)	0.70
Diabetes, *n* (%)	17(6.0)	12(6.1)	5(5.9)	1.00
CCD, *n* (%)	46(16.3)	30(15.2)	16(18.8)	0.55
CKD, *n* (%)	18(6.3)	12(6.1)	6(7.1)	0.96
History of smoking, *n* (%)	136(48.1)	99(50.0)	37(43.5)	0.38
**Renal artery involvement in CTA**				0.39
No, *n* (%)	144(50.9)	104(52.5)	40(47.1)	
Unilateral, *n* (%)	117(41.3)	77(38.9)	40(47.1)	
Bilateral, *n* (%)	22(7.8)	17(8.6)	5(5.9)	
SBP at admission (mmHg)	149.01 ± 25.55	149.41 ± 25.20	148.09 ± 26.47	0.69
DBP at admission (mmHg)	85.43 ± 15.32	85.19 ± 14.95	86.01 ± 16.22	0.68
EF (%)	60.65 ± 4.51	60.54 ± 4.71	60.90 ± 4.01	0.54
WBC (10^9^/L)	11.33 ± 3.89	11.20 ± 3.79	11.65 ± 4.13	0.37
HGB (g/L)	140.38 ± 20.31	140.80 ± 21.24	139.40 ± 18.06	0.60
PLT (10^9^/L)	209.09 ± 74.59	209.68 ± 76.10	207.73 ± 71.38	0.84
APTT (s)	31.25 ± 4.74	31.33 ± 4.48	31.08 ± 5.33	0.69
D dimer (ng/mL)	1,020.0(557.0, 2,572.5)	1,057.5(612.5, 2,572.5)	908.0(551.0, 2,528.0)	0.20
Serum kalium (mmol/L)	3.66 ± 0.43	3.67 ± 0.41	3.63 ± 0.46	0.48
Blood calcium (mmol/L)	2.22 ± 0.13	2.23 ± 0.12	2.21 ± 0.13	0.48
ALT (u/L)	25.4(19.435,34.645)	25.8(19.33, 35.83)	24.7(19.85, 32.10)	0.56
BUN (mmol/L)	5.9(4.77, 7.06)	5.92(4.89, 7.09)	5.55(4.55, 6.70)	0.07
UA (mmol/L)	344.97 ± 117.12	352.45 ± 115.99	327.54 ± 118.57	0.10
Baseline SCR (umol/L)	63.4(52.55, 75.25)	64.99(54.17, 77.55)	60.42(50.0, 71.70)	0.42
Proealcitonin (ng/mL)	0.06(0.04, 0.11)	0.06(0.04, 0.11)	0.05(0.03, 0.12)	0.13
IL-6 (pg/mL)	32.37(15.905, 63.17)	30.09(15.91, 61.75)	36.70(16.53, 70.63)	0.39
CTn I (ug/L)	0.012(0.012, 0.015)	0.012(0.012, 0.015)	0.012(0.012, 0.017)	0.55
Pericardial effusion, *n* (%)	15(5.3)	10(5.1)	5(5.9)	1.00
Pleural effusion, *n* (%)	65(23.0)	49(24.7)	16(18.8)	0.35
N-terminal pro BNP (ng/L)	124.0(54.25, 392.69)	115.0(51.16, 377.0)	152.0(64.1,393.0)	0.29
LAC (mmol/L)	1.5(1.1, 2.1)	1.6(1.1, 2.15)	1.4(1.1, 2.06)	0.46
PaO_2_/FiO_2_ (mmHg)	333.61 ± 105.59	333.02 ± 108.60	333.99 ± 98.84	0.88
**Intraoperative factors**				
Emergency operation, *n* (%)	59(20.8)	37(18.7)	22(25.9)	0.23
Total operation duration (mins)	90.0(75.0, 120.0)	90.0(70.0, 120.0)	105.0(75.0, 125.0)	0.25
Dose of contrast media (mL)	40.0(20.0, 60.0)	40.0(20.0, 60.0)	40.0(20.0, 60.0)	0.30
**Type of operation**				
Complex EVAR, *n* (%)	8(2.8)	5(2.5)	3(3.5)	0.94
Combined renal arteriography, n (%)	112(39.6)	81(40.9)	31(36.5)	0.57
**Postoperative factors**				
MVT (hours)	0.0(0.0, 5.0)	0.0(0.0, 4.5)	0.0(0.0, 5.0)	0.90
LOS in ICU (days)	5.0(9.0, 15.0)	5.5(1.0, 8.5)	5.0(1.0, 7.0)	0.23
**Perioperative drugs**				
RASI, *n* (%)	227(80.2)	163(82.3)	64(75.3)	0.23
Loop diuretics, *n* (%)	112(39.6)	78(39.4)	34(40.0)	1.00
Vasopressors, *n* (%)	70(24.7)	50(25.3)	20(23.5)	0.87
Statins, *n* (%)	51(18.0)	37(18.7)	14(16.5)	0.78
Antibiotics, *n* (%)	273(96.5)	193(97.5)	80(94.1)	0.29

EVAR, endovascular aneurysm repair; Complex EVAR is defined as requiring simultaneous repair of branches; Combined renal arteriography is defined as the addition of renal arteriography on the basis of main artery angiography.

### Model development

**Figure 4:**
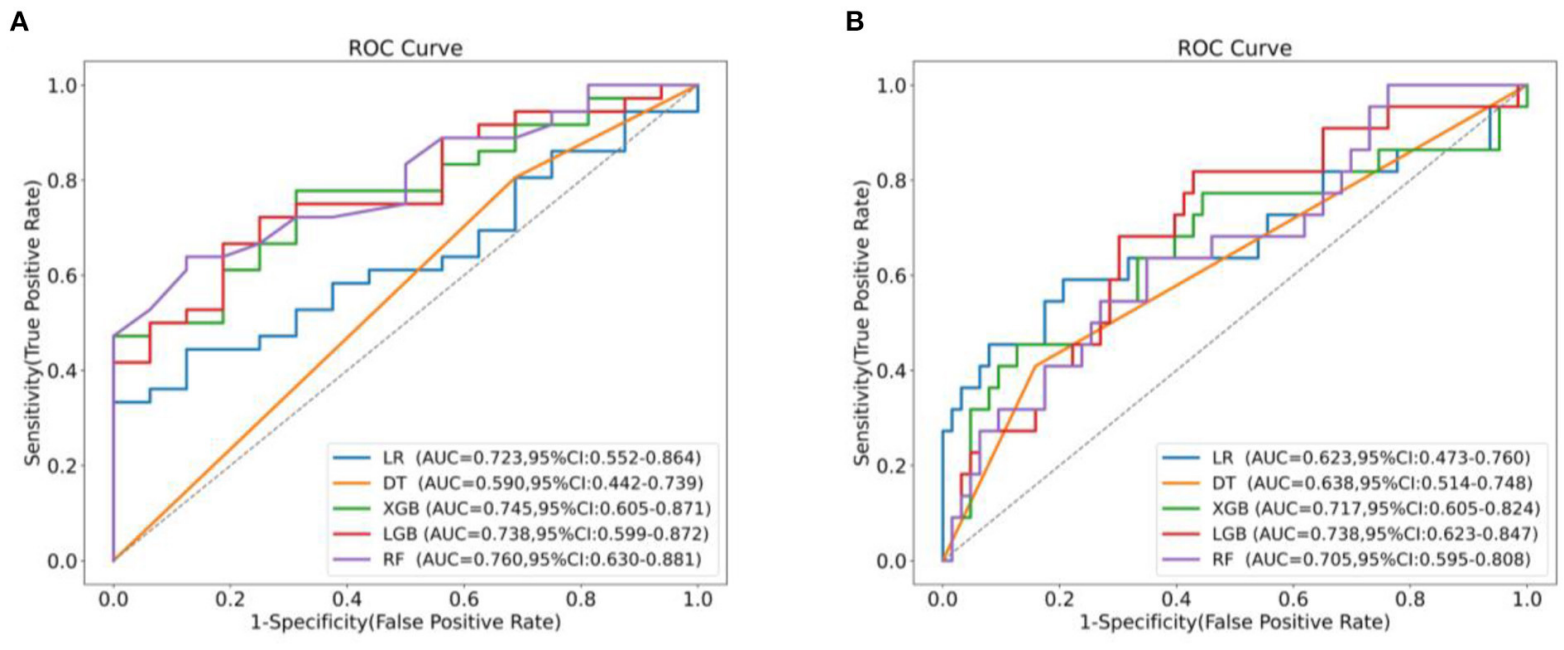
AUC comparison between machine learning models. **(A)** Shows the ROC curves and AUC values of different models predicting post-operative AKI after Bootstrap 1,000 times in the TAAAD training set. **(B)** Shows the ROC curves and AUC values of different models predicting postoperative AKI after Bootstrap 1,000 times in the TBAAD training set.

**Figure 5:**
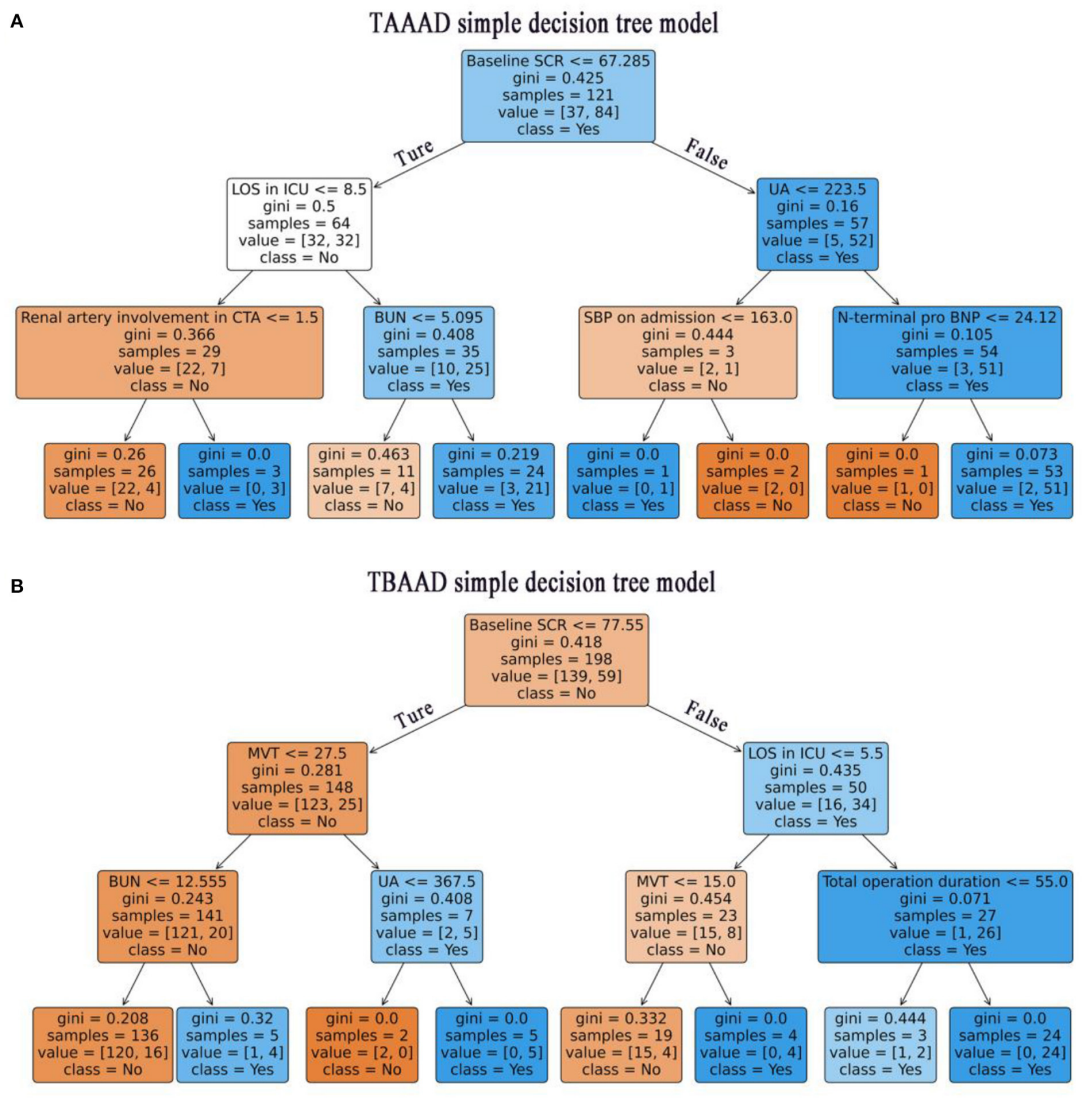
Visualization of the decision tree model. Recursive determination of whether a patient has AKI, blue represents AKI and orange represents Non-AKI. Each box represents a node and the straight lines with arrows represent edges, both on the left are Ture and both on the right are False. The main contents contained in the boxes are: the features used to slice the current node; The Gini represents the possible error rate of the current node, and the smaller the Gini index in this figure, the higher the color density of the node. Value is the actual number of non-AKI and non-AKI patients contained in the current node, and class represents the patient class predicted by the current node (class = No: non AKI patients, class = Yes: AKI patients). **(A)** Shows the TAAAD-AKI decision tree model. **(B)** Shows the TBAAD-AKI decision tree model.

We used LR, DT, RF, XGboost and LightGBM to construct the models, and used all features in the TAAAD and TBAAD training sets as input variables, respectively, and used Bootstrap (1,000 times) for internal validation in the training set to improve model stability. As shown in Figure 4, for TAAAD-AKI, the RF model exhibited the best prediction performance (AUC = 0.760, 95% CI:0.630–0.881), while for TBAAD-AKI, the LightGBM integrated learning model was the best (AUC = 0.734, 95% CI:0.623–0.847). It is worth mentioning that although DT may be too biased, it is clear and easy to understand and is the basis for learning and understanding other machine models. Figure 5 visualizes the process of DT recursively determining whether a patient has postoperative AKI from the initial root node to the final leaf node. As seen in Figure 5A, three of the eight leaf nodes in the TAAAD-AKI decision tree model have a Gini index of more than 0.2, and Figure 5B shows that four of the eight leaf nodes in the TBAAD-AKI model have a Gini index >0.2. Both of these suggest poor accuracy.

### Variable filter

**Figure 6:**
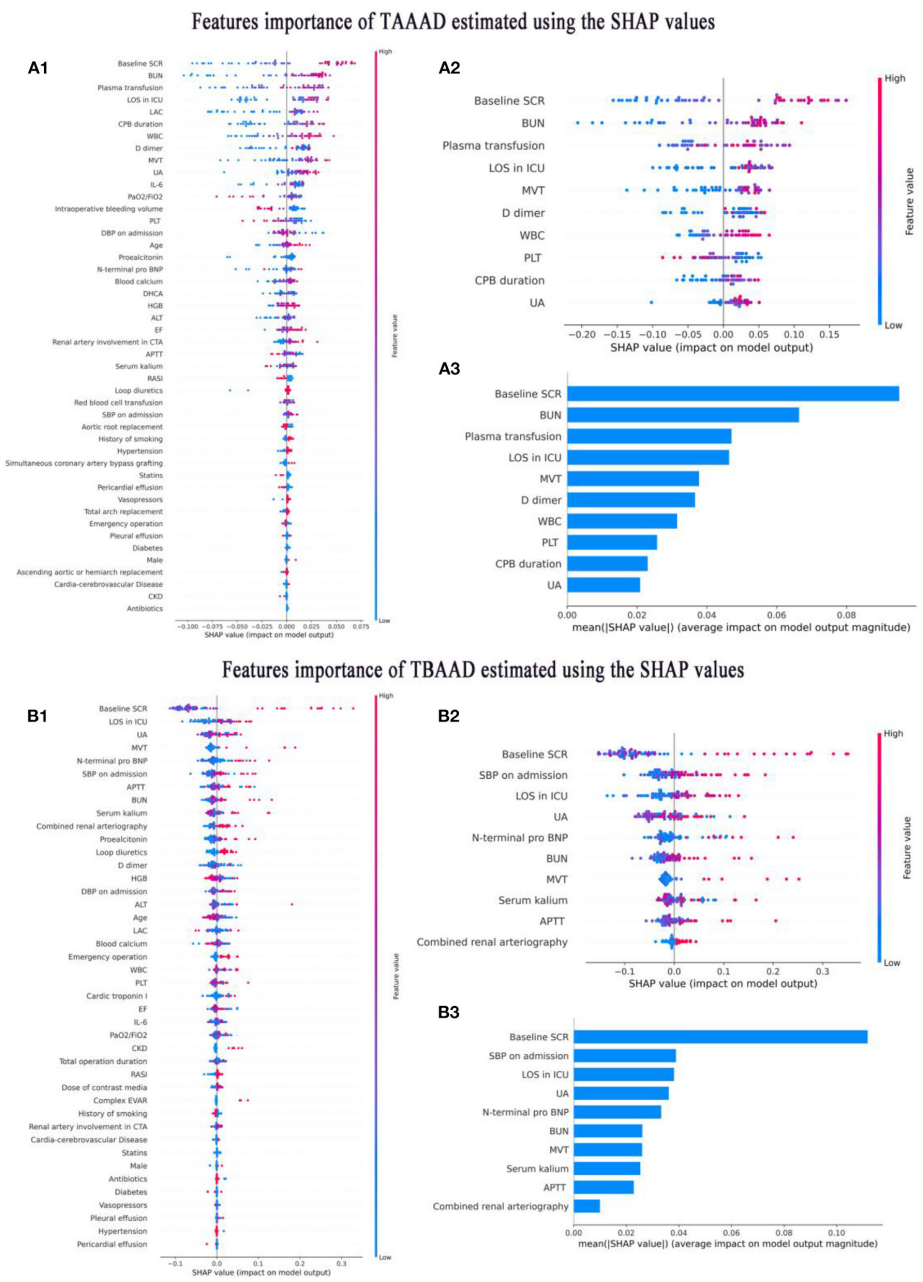
Interpretation of variable importance using SHAP values. SHAP assigns points to each feature of the patient in the graph, with features decreasing in importance from high to low, and colors representing the magnitude of the feature value (high in red, low in blue); the *X-*axis is used to measure the impact of the feature on AKI (positive on the right, negative on the left; the higher the value, the stronger the impact). **(A1–A3)** Shows the features importance of TAAAD-AKI. **(B1–B3)** Shows the features importance of TBAAD-AKI.

The best models based on TAAAD-AKI and TBAAAD-AKI were analyzed using the SHAP package for model interpretation, and the higher the SHAP value of a feature, the higher the likelihood of postoperative AKI. Figures 6A1,B1 show the descending ranking according to SHAP values after inclusion of all variables in the training set. To prevent overfitting and increase clinical controllability, we screened the variables by the REF method and selected 10 feature variables each by five-fold cross-validation. Figures 6A2,B2 show the effect of the compact model distribution explained by SHAP again after screening. Figures 6A3,B3 then show the importance ranking of the transformed predictor variables. We found that admission baseline SCR was the top-ranked predictor in both intact and compact models for patients with both types of entrapment. In both types of entrapment, the same common indicators of postoperative AKI in addition to baseline SCR are: admission BUN and UA, MVT and LOS in ICU. In TAAAD-AKI, the specific predictors are: WBC, PLT and D dimer at admission, Plasma transfusion and CPB duration in surgery. In the TBAAD-AKI prediction model, the specific predictors are: N-terminal pro BNP, Serum kalium, APTT and SBP at admission, Combined renal arteriography in surgery. After variable screening, we included the selected variables in the model and used Grid Search to determine the optimal hyperparameters. Hyperparameters were searched and visualized in TAAAD-AKI for the RF model and in TBAAD-AKI for the LightGBM model (see Tables 3, 4 for specific hyperparameter settings).

**Table 3:** TAAAD-AKI random forest.

**Hyperparameters**	**Search domain**	**Final setting**
max_depth	10–200	10
max_features	“auto”, “sqrt”	auto
min_samples_leaf	1,2,4,8	8
min_samples_split	2,5,10	10
n_estimators	1–200	90

**Table 4:** TBAAD-AKI LightCBM.

**Hyperparameters**	**Search domain**	**Final setting**
num_leaves	5–31	5
Max_depth	3,4,5	3
subsample	0.8,0.9,1.0	0.8
Colsample bytree	0.8,0.9,1.0	0.8
reg_alpha	np.log(0.01), np.log(1,000)	6.9
reg_lambda	np.log(0.01), np.log(1,000)	6.9

### Model validation and evaluation

**Figure 7:**
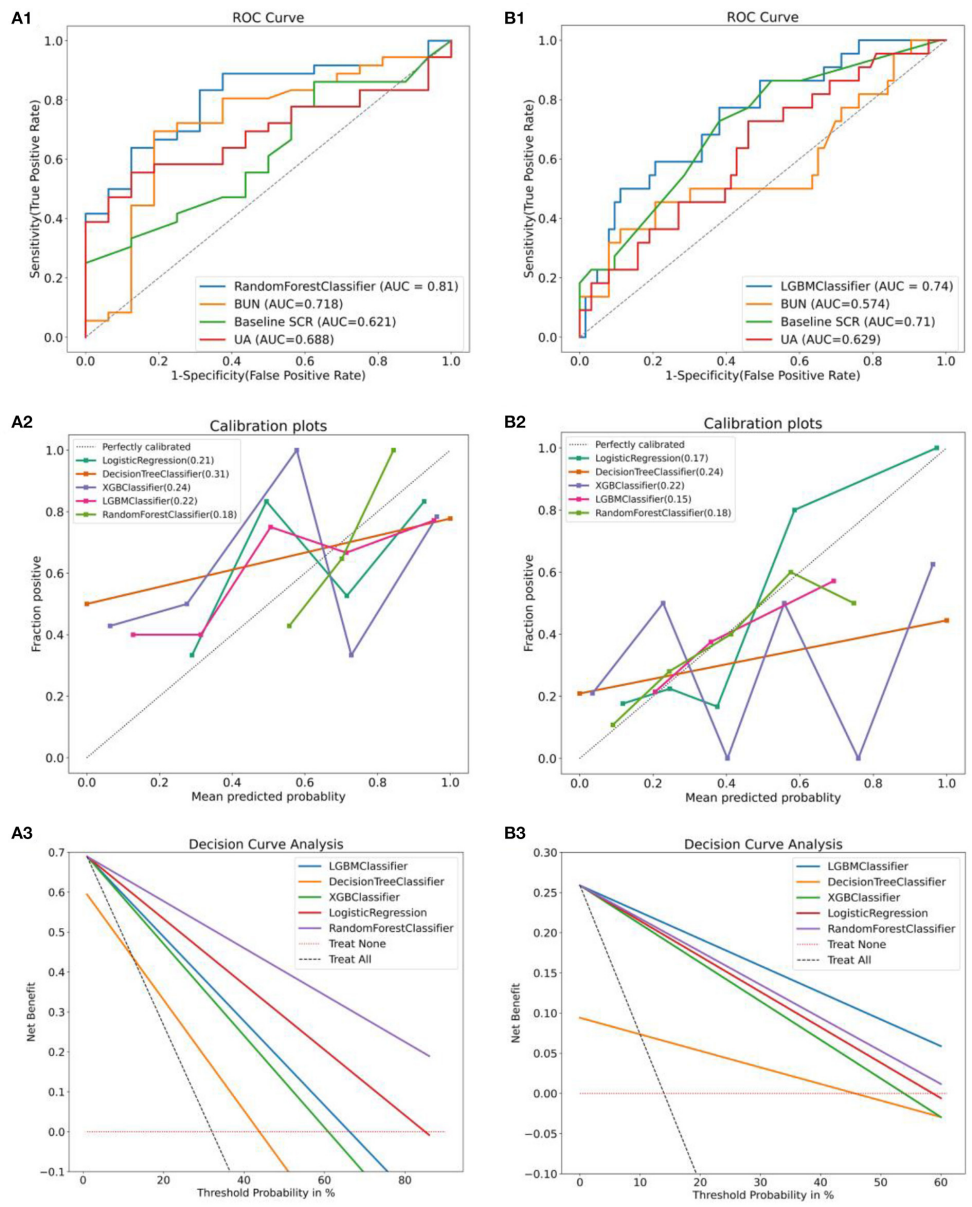
Evaluation and validation of the two models. **(A1,B1)** are comparisons of the performance of the compact model after feature selection with the prediction performance of AKI occurrence in the test set using baseline SCR, BUN and UA alone. **(A2,B2)** are the calibration curves for different machine learning models and the Brier scores. **(A3,B3)** are the clinical decision curves for the different machine learning models. The gray dashed line is the benefit rate for all patients who received the intervention, and the pink dashed horizontal line is no benefit for all patients who did not receive the intervention. The intersection with all is taken as the starting point and the intersection with None as the ending point, within which is the corresponding total net benefit.

We validated the constructed compact ML prediction models in the test set. Figures 7A1,B1 show the performance of two types of AAD using the best ML compact prediction model to compare with the AKI clinically sensitive measures SCR, BUN and UA to predict AKI, respectively. We found that the combined prediction level of machine learning is higher than that of a single predictor. Figures 7A2,B2 show the calibration curves of the different machine learning models for the two types of AAD, respectively, and it can be clearly seen that in TAAAD-AKI, the calibration curve of RF fits the ideal curve (diagonal) the best and has the smallest Brier score (0.16). That is, the predicted value of RF differs the least from the true value compared to the other models. In contrast, the calibration curve of LightGBM in TBAAD-AKI is the closest to the ideal curve and has the smallest Brier score (0.15). The prediction consistency of the model was likewise recognized. Figures 7A3,B3 show the analysis of decision curves for different machine learning models. Similarly, RF showed the greatest net clinical benefit in the TAAAD-AKI test set; LightGBM showed the widest range of benefit in TBAAD-AKI, meaning that the model has a higher clinical utility.

## Discussion

In this study, we retrospectively collected perioperative data of patients undergoing AAD surgery in our hospital for three consecutive years and found that the incidence of AKI was higher in both TAAAD and TBAAD patients than in other studies, which may be related to our improved method of assessing baseline SCR. Because this improves the sensitivity of AKI diagnosis and is more beneficial in detecting patients with early AKI compared to routine admission SCR as a baseline value. In addition, this study constructed clinical prediction models for AAD-AKI with different typing using the ML algorithm with superior performance. The results illustrate that the best model is not consistent across different data and relying on empirical selection of a fixed single model may reduce its reliability. Our results show that several clinically common indicators of kidney function, such as baseline SCR, hospital admission BUN and UA, were significantly different in both AKI and non-AKI groups in the TAAAAD and TBAAD whole datasets. Furthermore, baseline SCR, hospitalized BUN, and UA were also important predictors of both types of AAD-AKI in the best ML models constructed using training set data, respectively. SCR is a metabolite of skeletal muscle phosphoric creatine, BUN is a metabolite of proteins, and UA is a metabolite of purines, both of which are excreted through kidney urine, and urination disorders lead to varying degrees of upregulation. In addition, patients with AAD are more likely to develop hypertension, and hyperuric acid is often associated with hypertension (36). Hyperuric acid crystallization in kidney tissue resulting in obstructive kidney injury is also one of the pathogenesis of AAD-AKI, and studies have shown that hyperuric acid is associated with AKI incidence and death outcomes in patients (37). Although they are interfered with by a variety of factors and are not perfect indicators of kidney function, they are still a major marker for diagnosis of AKI under existing conditions. Perioperative dynamic monitoring of these early warning molecules is particularly important for early detection of AKI.

Other predictors in the TAAAD-AKI compact model have been extensively discussed in several studies. Decreased PLT on admission and increased D dimer are positively associated with the development of AKI and may be associated with more severe disease. When blood flow passes through a non-endothelialized pseudolumen, it triggers the activation of platelets and the coagulation system, leading to platelet depletion and an increase in fibrin degradation products (38). CPB, as a marker of surgical complexity, has been repeatedly demonstrated as a risk factor for the development of TAAAD-AKI. The main mechanisms leading to AKI are (1) ischemia-reperfusion injury (IRI). Low pressure, low flow, non-pulsatile perfusion, hemodilution, and massive intraoperative bleeding during CPB can lead to renal hypoperfusion (39). (2) Inflammation and oxidative stress. The contact of blood with artificial materials activates immune cells and inflammatory factors during extracorporeal circulation transfer (40). In addition, exposure of erythrocytes to non-physiological ducts and shear forces of blood flow can lead to hemolysis, production of large amounts of free HB and oxygen radicals, and even inflammatory storms. WBC is one of the markers of inflammatory response, and this study suggests that elevated WBC leads to an increased incidence of postoperative AKI, which is consistent with the findings of Takahashi, a Japanese scholar (41). The TAAAD surgical procedure bleeds heavily and always requires transfusion of blood products. However, blood transfusion can increase the inflammatory response. In CPB patients, blood transfusion has been shown to be associated with AKI and RRT (8, 9, 42). TAAAD patients all require ventilator-assisted ventilation for varying periods of time after deep anesthesia. Prolonged MVT is an important risk factor for the development of AKI, which is consistent with the results of other studies (11). We concluded that: continuous optimization of the procedure, shortening of CPB time, optimization of volume status, and use of goal-directed therapy (GDT) (43); Reducing traumatic bleeding, using Autologous platelet-rich plasma (APRP) to reduce intraoperative allogeneic blood transfusion; promoting the recovery of patients' autonomic respiratory function as soon as possible, stopping mechanical ventilation at the right time, and reducing LOS in ICU can help reduce the risk of AKI in TAAAD patients after surgery.

The characteristic predictors in the compact model of TBAAD-AKI are equally worthy of discussion. As with TAAAD, LOS in ICU and the accompanying prolonged MVT are also associated with TBAAD-AKI. On the one hand, MVT indirectly reflects the severity of lung injury. and that respiratory insufficiency leads to hypoxemia, and the kidney, as an oxygen-sensitive organ, is therefore susceptible to induce AKI. On the other hand, positive pressure ventilation leads to a decrease in cardiac output and causes inadequate renal perfusion which can also lead to AKI. prolonged APTT is considered to be related to the large amount of thrombin depletion during thrombosis of the patient's pseudocavitary blood. All of these factors suggest that the patient is relatively sicker and more likely to develop AKI. Elevated Nt-proBNP on admission is one of the specific indicators of cardiac insufficiency, and low cardiac output causes a decrease in glomerular filtration rate (GFR), which is also described as “Cardio-renal Syndrome” (CRS) (44). It is also elevated during the oliguric phase of AKI due to reduced excretion of Nt-proBNP. Poorly controlled hypertension is thought to be the main cause of morbidity in patients with AAD. In this study, elevated admission SBP was found to be a predictor of the development of AKI after TBAAD. Luo et al. (5) considered that admission systolic blood pressure >140 mmHg was an independent risk factor for AKI after EVAR. Excessive blood pressure will further cause false lumen extension, eventually leading to the occurrence of AKI. EVAR in patients with TBAAD requires a large amount of contrast media to determine the location of the breach, guide wire and stent. Contrast-induced acute kidney injury (CI-AKI) is significantly increased with the use of large amounts of contrast media in a short period of time (45). EVAR in patients with TBAAD requires a large amount of contrast media to determine the location of the breach, guide wire and stent. Contrast-induced acute kidney injury (CI-AKI) is significantly increased with the use of large amounts of contrast media in a short period of time (46). Contrast dose was also not included in our study, but when we defined combined renal arteriography as adding renal arteriography to aortography, it was found to be positively associated with TBAAD-AKI. We believe that when the kidneys are more heavily impacted by contrast media, it is most likely to be a risk factor for CI-AKI, although more prospective evidence is needed to support our view. In summary, for TBAAD patients, we believe that more stringent blood pressure control, protection of cardiac function, avoidance of exposure to nephrotoxic drugs, timely postoperative hydration therapy, reduction of unnecessary mechanical ventilation, and active correction of complications are of great significance for the diagnosis and treatment of AKI.

In summary, we found differences in the incidence, important risk predictors and renal protective measures between TAAAD-AKI and TBAAD-AKI, which are ultimately related to their pathogenesis. In our subsequent related studies, we found that the pathophysiology of AKI due to the two types of entrapment differs in terms of ischemia-reperfusion injury, inflammation and oxidative stress, activation of neurohumoral fluids, obstruction of metabolic substances, and endogenous and exogenous nephrotoxins. A more detailed description requires our further confirmation.

## Strengths and limitations

The strengths of our study are: (1) We adjusted the ambiguous baseline SCR assessment method according to the pathogenic characteristics and clinical experience of AAD patients. (2) We simultaneously constructed and compared clinical prediction models of two types of AAD-AKI, which have not been found in other literatures so far. (3) According to the actual situation of the two types of AAD datasets, we select the best ML model that matches them, instead of using the same machine algorithm without verification.

Our study also has some limitations: (1) We are still limited by the problem of insufficient sample. The larger the sample of the prediction model, the better the effect of machine learning. (2) There are a large number of missing values in important variables such as BMI, urine protein, cystatin C, and urine volume, so they were not included in the study, which may reduce the performance of the model. (3) Our research also requires external validation to determine the robustness of the model.

## Conclusion

In this study, we successfully constructed and validated a clinical prediction model for postoperative AKI in TAAAD and TBAAD patients using different machine learning algorithms. ML is more accurate than the traditional logistic regression model. With the popularization of big data and artificial intelligence, the application of machine learning in the medical field will be more extensive. After variable screening, we found that the predictors of AKI caused by the two types of AAD were very different, and the corresponding treatment strategies were also different. These findings need to be further confirmed in future prospective studies.

## Data availability statement

The original contributions presented in the study are included in the article/Supplementary material, further inquiries can be directed to the corresponding author.

## Ethics statement

The studies involving human participants were reviewed and approved by the Ethics Committee of the First Affiliated Hospital of Xinjiang Medical University. Written informed consent for participation was not required for this study in accordance with the national legislation and the institutional requirements.

## Author contributions

LS and LX designed the study. WZ and PK were in charge of machine learning analysis. CS, CX, and JX were responsible for data collation and statistical analysis. LX and HX wrote the first draft. LS reviewed and checked the manuscript. All authors contributed to the article and read and approved the submitted version of the final manuscript.

## Funding

This work was supported by the National Natural Science Regional Fund (81860125) and the Postgraduate Innovation and Entrepreneurship Project of Xinjiang Medical University (CXCY2021004).

## Conflict of interest

The authors declare that the research was conducted in the absence of any commercial or financial relationships that could be construed as a potential conflict of interest.

## Publisher's note

All claims expressed in this article are solely those of the authors and do not necessarily represent those of their affiliated organizations, or those of the publisher, the editors and the reviewers. Any product that may be evaluated in this article, or claim that may be made by its manufacturer, is not guaranteed or endorsed by the publisher.
